# Deformable cardiac surface tracking by adaptive estimation algorithms

**DOI:** 10.1038/s41598-023-28578-0

**Published:** 2023-01-25

**Authors:** E. Erdem Tuna, Dominique Franson, Nicole Seiberlich, M. Cenk Çavuşoğlu

**Affiliations:** 1 Department of Electrical, Computer, and Systems Engineering, Case Western Reserve University, Cleveland, OH 44106 USA; 2 Department of Biomedical Engineering, Case Western Reserve University, Cleveland, OH 44106 USA; 3 Department of Radiology, Michigan Medicine, University of Michigan, Ann-Anbor, MI 48109 USA

**Keywords:** Biomedical engineering, Electrical and electronic engineering

## Abstract

This study presents a particle filter based framework to track cardiac surface from a time sequence of single magnetic resonance imaging (MRI) slices with the future goal of utilizing the presented framework for interventional cardiovascular magnetic resonance procedures, which rely on the accurate and online tracking of the cardiac surface from MRI data. The framework exploits a low-order parametric deformable model of the cardiac surface. A stochastic dynamic system represents the cardiac surface motion. Deformable models are employed to introduce shape prior to control the degree of the deformations. Adaptive filters are used to model complex cardiac motion in the dynamic model of the system. Particle filters are utilized to recursively estimate the current state of the system over time. The proposed method is applied to recover biventricular deformations and validated with a numerical phantom and multiple real cardiac MRI datasets. The algorithm is evaluated with multiple experiments using fixed and varying image slice planes at each time step. For the real cardiac MRI datasets, the average root-mean-square tracking errors of 2.61 mm and 3.42 mm are reported respectively for the fixed and varying image slice planes. This work serves as a proof-of-concept study for modeling and tracking the cardiac surface deformations via a low-order probabilistic model with the future goal of utilizing this method for the targeted interventional cardiac procedures under MR image guidance. For the real cardiac MRI datasets, the presented method was able to track the points-of-interests located on different sections of the cardiac surface within a precision of 3 pixels. The analyses show that the use of deformable cardiac surface tracking algorithm can pave the way for performing precise targeted intracardiac ablation procedures under MRI guidance. The main contributions of this work are twofold. First, it presents a framework for the tracking of whole cardiac surface from a time sequence of single image slices. Second, it employs adaptive filters to incorporate motion information in the tracking of nonrigid cardiac surface motion for temporal coherence.

## Introduction

The magnetic resonance imaging (MRI) guided interventions are getting widespread applications in clinical settings due to MRI’s high soft tissue contrast and radiation-free imaging. MRI catheterization is one such emerging technology, where CMR is being used for guiding catheters for diagnostic and interventional purposes^1^. MRI-guided diagnostic cardiac catheterization is employed for accurately measuring the pulmonary vascular resistance. MRI-guided targeted interventional cardiac procedures includes catheter ablation for the treatment of ventricular tachycardia and atrial fibrillation^2^.

The diagnostic MRI-guided catheter procedures require accurate cardiac segmentation, which provides delineation of the cardiac boundaries, where this boundary information has been widely utilized in the development of global and regional quantitative indices, which help to distinguish between pathological and healthy subjects in clinical practice^3–5^. During the interventional CMR procedures accurate and real-time tracking of the cardiac surface from image data is needed for surgical planning as well as reliable ablation of the desired target area via navigating a manual or a robotic instrument^6^. All these procedures require the modeling and computation of the cardiac surface deformations as heart goes through a nonrigid motion.

This work presents a method for modeling and tracking the cardiac surface deformations via a low-order probabilistic model with the future goal of utilizing this approach for the interventional cardiac procedures under MR image guidance. The goal is to model the shape and surface of the heart, so that the proposed approach could be employed in targeted cardiac interventions such as cardiac catheter ablation procedures, where the non-periodic motion of the heart is critical. For this purpose, it incorporates motion information in the surface tracking to make the algorithm more robust to rapid and dynamic cardiac shape changes for the cases when the heart motion statistics change abruptly and significantly, such as during arrhythmias. The aim of the presented study is not to utilize discrete measurements to extract and track individual features, hence it is not intended for diagnostic procedures that prioritize clinical measurements, such as inferring ejection ratios of the ventricles, strain values, and other quantitative indices.

Object tracking is a challenging task with numerous applications in computer vision, where given the initialized state such as the location and the size of an arbitrary target of interest in a frame of a video, the aim of tracking is to estimate the states of the target in the subsequent frames the best possible accuracy^7^. Much progress has been made in recent years, where deep learning and correlation filter based approaches have gained increasing attention^8–11^. Lately, there has been a growing interest in applying machine and deep learning based methods to cardiac motion segmentation and tracking problems^12–15^. The clinical applications of these techniques has been primarily focused on the diagnostic cases to assess cardiac function such as by providing accurate estimation of the right and left ventricular volumes, ejection ratios, and other quantitative indices, which is not the focus of this study. A recent study^16^ compares various CMR software packages for such purposes.

Modeling deformation has become a key research topic in medical image analysis^17^, surgical simulation^18,19^, medical image registration^20,21^, cardiac motion recovery^22–25^, cardiac image segmentation and functional analysis^3–5,26,27^.

Traditional cardiac motion segmentation and tracking approaches can be broken down into^4^ image based^28^, classification based^29^, and deformable model based^30^ approaches. Deformable models; snakes^31^, level-set evolution^32^, and its variants^33,34^, have been extensively applied to the ventricle tracking and segmentation problems. They are effective tools for cardiac motion reconstruction. Although the term *deformable models* originally referred to active contours/snakes presented by Kass *et al.*^31^, in this study it is used for their extensions to surface and volumetric models with superquadrics^35^. A concise introduction to deformable models, its extensions, and applications to medical image segmentation can be found in^33^.

Chen *et al.*^36^ applied superquadrics with tapering and bending deformations to model the left-ventricle (LV) for image segmentation and shape analysis. Deformable models with parameter functions are presented in^37,38^ to analyze the LV motion. Haber *et al.*^39^ extended parametric functions to recover the right-ventricle (RV) motion, and Park *et al.*^40,41^ used deformable models for RV-LV modeling and conducting 4D cardiac functional analysis via finite element modeling (FEM). More recently, Wang *et al.*^42^ introduced meshless deformable models for 3D cardiac motion and strain analysis from tagged MRI.

Incorporating priors is an important aspect of solving cardiac segmentation and tracking problems. The use of priors such as shape, motion, or texture could aid in these tasks by increasing their robustness and accuracy^3^. Integrating shape priors has been widely studied, whereas using motion information has taken less attention, partly due to the complexity and the variability of the heart motion but also solely using end-diastole (ED) and end-systole (ES) image segmentations are sufficient for estimating cardiac diagnostic functions in clinical practice^4^. Various previous work studied motion prior in the context of spatiotemporal atlases of cardiac motion^43–46^.

Motion prior has different purpose respectively in segmentation and tracking problems. In the segmentation problem, the goal is the delineation of the surface boundaries in each image and thus utilizing motion information introduces temporal coherence in the extracted borders. In contrast, the tracking problem aims to recover the trajectories of the target material points on the cardiac surface, which is essential in the image-guided robotic interventions, where motion information provides temporally consistent trajectories^47^.

A number of previous studies tried to incorporate motion information into cardiac tracking. If minimum temporal information is available, then a weak temporal prior information could be integrated via temporal position averaging^47^. In contrast, information regarding expected cardiac motion could be incorporated as a strong prior. Sequential approach^48–51^ propagates the results from previous time step as the initialization for the current time step, which models cardiac motion as a Brownian process. Other approaches^52,53^ try to learn more complex heart dynamics from a prior training set.

Sequential approach assumes no prior knowledge regarding the temporal dynamics of the heart, whereas learning-based approaches are limited to the information provided in the training set. Such assumptions would be insufficient in the case of arrhythmia, in which heart dynamics goes through abrupt changes.

Utilizing motion priors in cardiac surface tracking in the context of catheter ablation procedures is more challenging due to significant changes in heart dynamics during arrhythmia. In^54–56^, authors previously showed feasibility of employing recursive adaptive filters for tracking point-of-interest motion on cardiac surface respectively under arrhythmia and normal conditions during beating heart surgery. McEachen^57^ presented a method to track the LV endocardial contour via recursive adaptive filters.

Adaptive filters used in this study have a robustness trait that makes their output (i.e. predicted trajectory) less susceptible to disturbances from irregular heart dynamics during arrhythmia^55^, thus they could incorporate motion information smoother and address the shortcomings of the aforementioned approaches.

This paper presents the cardiac surface tracking as a state estimation problem via Bayesian formulation. Particle filter based belief propagation facilitates incorporating temporal motion information of the heart. Cardiac surface motion is represented as a dynamic system and parameterized by a low-order deformable model, which constitutes the system state. Deformable models provide a versatile framework to introduce shape prior to control the extent of the deformation. Dynamic model of the system uses adaptive filters to address modeling of complex heart motion. Utilizing adaptive filters allows to introduce a priori knowledge about the active cardiac motion and thus to recover some movement (like the tangential motion), which cannot be obtained from classical geometrical tracking methods. Particle filters (or Sequential Monte Carlo methods)^58^ employed in this study provides a framework for incorporating the uncertainty into tracking via Markov assumption by only taking information into account from the previous time step to estimate the pose of the object at the current time step. This makes such an approach suitable for time-critical, online applications such as targeted cardiac catheter ablations. The main contribution of the presented work is incorporating the prior cardiac motion information via adaptive filters into the particle filter based tracking of the cardiac surface for targeted cardiac catheter ablations, in contrast to the tracking by detection methods^59^, which independently detect object and its pose at each time step.

The approach presented here is not limited to modeling the deformation of a particular cardiac chamber. Due to more common availability of the validation data, a low-order deformable surface model is applied to recover the biventricular geometry and deformations. The deformable model framework was originally developed in^60^ and used for biventricular modeling in^40^. Here, it is embedded in the particle filter formulation. An adaptive filter is used in the motion update step of the particle filter. It introduces temporal coherence to the tracked cardiac surface trajectory. Image slice plane and deformable model intersection yields the measurement model. Tracking procedure is validated by a numerical phantom and multiple real cardiac MRI datasets. The approach presented here is not limited to a specific type of MRI modality *i*.*e*.  cine MRI, tagged MRI, or phase contrast MRI^4^. In this study, cine MRI datasets are used for validation. A list of studies which explore tracking of local myocardial deformations by utilizing tagged and phase contrast MRI modalities can be found in^22,61–63^.

Another contribution of this work is the tracking of whole cardiac surface from a time sequence of single 2D image slices. Previous works used a single slice to track a specific cardiac contour, utilized either a stack of slices, or volumetric data for the recovery of surface or volumetric motion.

The presented cardiac surface tracking approach based upon several assumptions. First, the twisting motion is essential in ventricular ejection. If ejection was simply the result of contraction of myocardial fibers, the ejection fraction would be 15–20%, whereas the actual ejection fraction of the human heart is 60–70%. This is due to the twisting of myocardial fibers^64^; hence twisting can not be ignored while modeling cardiac surface deformations. Here, interventricular septum rotation^40^ is used to recover the twisting motion by assuming LV and RV have similar twisting patterns^65,66^.

Second, the myocardium is an almost incompressible material. Its constituents are mainly composed of water, which is almost perfectly incompressible. Yet, the myocardium is perfused with blood, which affects the its total volume over the cardiac cycle. A few studies^67–69^ have been carried out to quantify the myocardial volume change over the cardiac cycle. The common conclusion was the total myocardial volume changes no more than 4$\%$ during a cardiac cycle, meaning the myocardium is not perfectly incompressible. However, this volume change is distributed in all three directions. Thus, the parametrized cardiac surface deformations described in  “Dynamic system state” section are volume preserving. As the volume change is distributed in all three directions, *i*.*e*. the myocardial wall thickening would result in longitudinal shortening during systole, this makes it feasible to track cardiac surface via a single image slice. Previous studies utilized the incompresibility of the myocardium^70–72^.

Additionally, RV wall is three to six times thinner than the LV, reaching the limit of MRI spatial resolution^5^. Thus, only endocardium of RV is considered, whereas both endocardium and epicardium of LV are modeled.

Finally, the image slices employed in the tracking algorithm assumed to be presegmented; meaning boundaries of the RV and LV walls are already delineated when they are used in the measurement update step of the particle filter.

## Results

The feasibility of the presented approach is initially shown on a numerical phantom with known parameters. Then, it is validated with multiple real cardiac MR datasets, each representing a single cardiac cycle.

**Figure 1:**
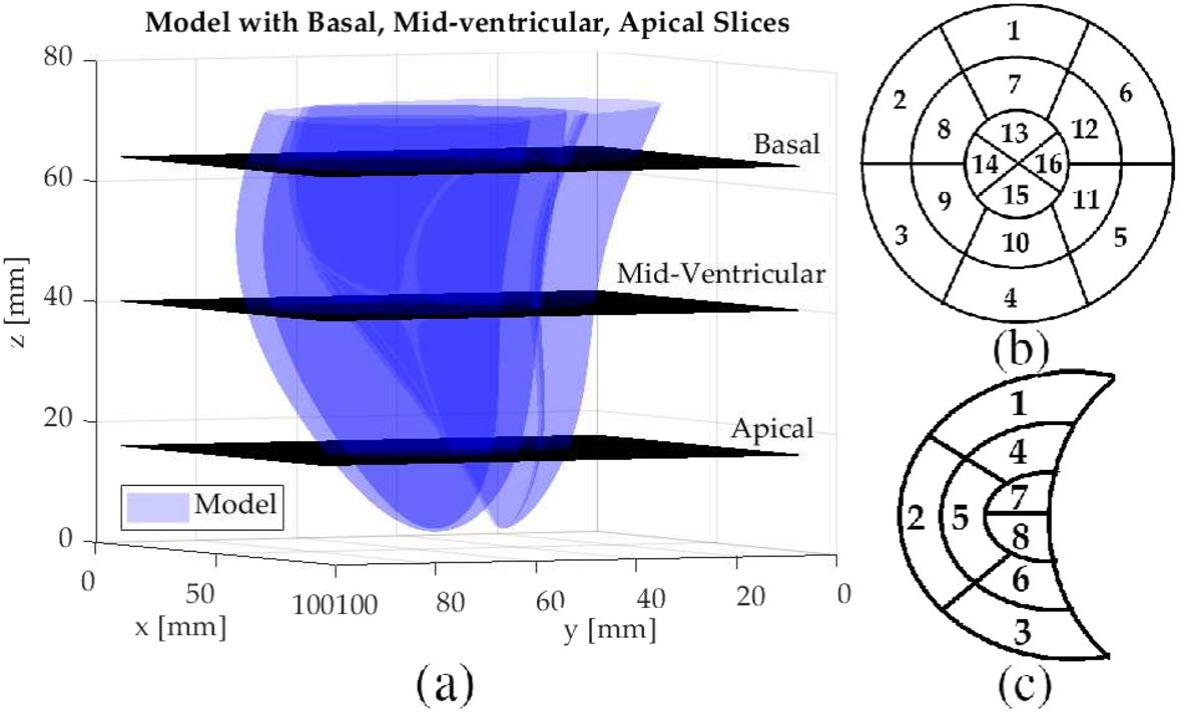
(**a**) The intersection of the biventricular cardiac model and three MR slice planes corresponding to the basal, mid-cavity, and apical sections. (**b**) 16-point LV model. (**c**) 8-point RV model. The POI locations of the LV and RV segments on the cardiac surface, identified by the indexes in the corresponding 16-point and 8-point models are as follows: *Anterior*: LV1, LV7, LV13, RV1, RV4, RV7; *Anteroseptal*: LV2, LV8; *Septal*: LV14; *Inferoseptal*: LV3, LV9; *Inferior*: LV4, LV10, LV15, RV3, RV6, RV8; *Lateral*: LV16, RV2, RV5; *Inferolateral*: LV5, LV11; *Anterolateral*: LV6, LV12.

The tracking performance is evaluated for points-of-interest (POIs) on the cardiac surface. The POIs are determined via the intersection of the deformable biventricular model and three MR slice planes corresponding to the basal, mid-cavity, and apical sections (Fig. 1a). For the LV, a total of thirty-two points are selected, sixteen points each for endocardium and epicardium, based on the 16-point LV model^73^; six points for the basal, six points for the mid-ventricular, and four points for the apical slices (Fig. 1b). There is no standard model for RV segmentation and several different models have been proposed in previous studies^66^. An 8-point RV model was chosen for the RV endocardium to evaluate the tracking performance^74^ (Fig. 1c).

### Simulation results

The tracking results for the numerical phantom are given respectively in Tables 1 and 2 for the 16-point LV epicardium and endocardium models, and in Table 3 for the 8-point RV model. The root-mean-square (RMS) tracking errors and the standard deviations are reported.

The tracking errors for the numerical phantom is overall within 2 pixels (3 mm). The RMS errors are respectively 0.97 mm for the POIs located on LV and 1.56 mm for the POIs located on RV. The overall RMS error is 1.11 mm. The proposed algorithm was able to follow the motion of POIs located on LV more precisely than the POIs located on RV. Additionally, for both LV and RV, POIs located on the basal and mid-ventricular sections were tracked more accurately.

**Table 1:** RMS tracking errors of the numerical phantom for the 16-point LV model for the LV epicardium.

Plane location	POI Index					
	RMS tracking error [mm]					
	(Std. Dev.) [mm]					
	LV1	LV2	LV3	LV4	LV5	LV6
Basal	0.79	1.10	1.12	0.68	0.83	1.03
	(0.12)	(0.21)	(0.15)	(0.04)	(0.12)	(0.12)

	LV7	LV8	LV9	LV10	LV11	LV12
Mid-ventricular	0.92	0.95	0.73	0.81	0.82	0.55
	(0.15)	(0.16)	(0.05)	(0.13)	(0.11)	(0.16)

	LV13	LV14	LV15	LV16		
Apical	2.65	2.17	1.69	2.74		
	(0.41)	(0.56)	(0.15)	(0.28)

**Table 2:** RMS tracking errors of the numerical phantom for the 16-point LV model for the LV endocardium.

Plane Location	POI Index					
	RMS tracking error [mm]					
	(Std. Dev.) [mm]					
	LV1	LV2	LV3	LV4	LV5	LV6
Basal	0.76	0.98	1.11	0.72	0.82	1.01
	(0.12)	(0.18)	(0.15)	(0.04)	(0.11)	(0.09)

	LV7	LV8	LV9	LV10	LV11	LV12
Mid-ventricular	0.82	1.65	1.07	0.66	1.67	1.20
	(0.10)	(0.15)	(0.11)	(0.11)	(0.18)	(0.11)

	LV13	LV14	LV15	LV16		
Apical	2.47	1.61	1.56	2.42		
	(0.30)	(0.60)	(0.27)	(0.18)

**Table 3:** RMS tracking errors of the numerical phantom data for the 8-point RV model.

Plane location	POI index		
	RMS tracking error (Std. Dev.) [mm]		
	RV1	RV2	RV3
Basal	1.86 (0.08)	0.29 (0.05)	0.26 (0.10)

	RV4	RV5	RV6
Mid-ventricular	0.42 (0.12)	0.30 (0.13)	0.29 (0.10)

	RV7	RV8	
Apical	1.70 (0.09)	3.58 (0.11)	

### Experiment results

**Figure 2:**
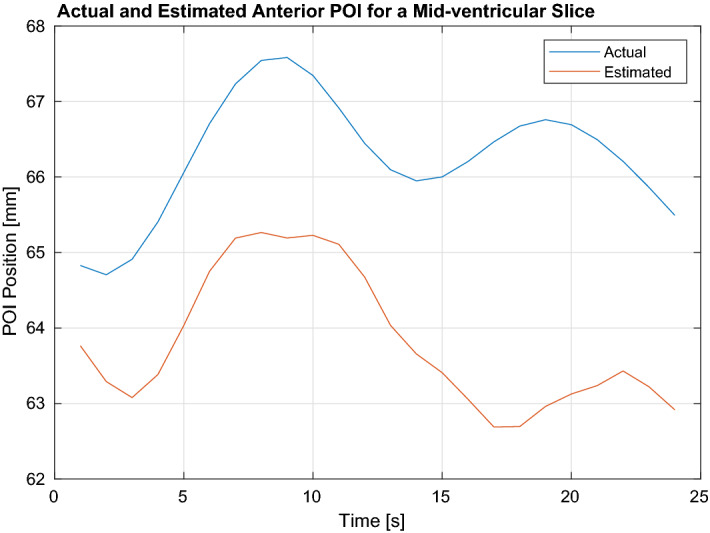
Shows the tracking results for an anterior POI position for a mid-ventricular slice.

The tracking results for the first experiment are presented respectively in Table 4 and in Table 5 for the 16-point LV epicardium and endocardium models, and in Table 6 for the 8-point RV model. First, the RMS tracking errors of the 32-point LV and 8-point RV models are computed for each of the eight datasets. Then, the averages of these RMS tracking errors across all the eight datasets are calculated and reported together with the corresponding standard deviations. The effect of integrating the twisting motion to parameterized deformations is also investigated. The results show the tracking errors for both cases when the twisting motion included as well as excluded from the parameterized deformations. Figure 2 shows the tracking result of an anterior POI position for a mid-ventricular slice.

The results show that with the fixed slice plane, the tracking errors are within 3 pixels (5.3 mm). The RMS errors are respectively 2.30 mm for the POIs on LV and 2.77 mm for the POIs on RV. The overall RMS error is 2.37 mm. The error values are not uniform across the surface and changes based on the POI location; indicating the deformations are not uniform across the cardiac surface. The model was able to track POIs located on basal and mid-ventricular sections more accurately compared to apical sections. Additionally, the tracking accuracy for the LV is higher than RV, which shows the parameterized deformations capture the uniform shape of the LV better than the nonuniform shape of the RV.

**Table 4:** The summary of the results for the 16-point LV epicardium model.

Plane Location	Model	POI Index					
		Average RMS Tracking Errors [mm]					
		(Std. Dev.) [mm]					
		LV1	LV2	LV3	LV4	LV5	LV6
Basal	Twist	2.35	3.12	2.18	2.13	2.35	2.88
		(0.66)	(1.29)	(1.44)	(0.82)	(1.65)	(1.58)
	No Twist	2.68	3.70	2.64	2.76	2.62	3.02
		(0.64)	(1.43)	(1.34)	(1.28)	(1.72)	(1.77)

		LV7	LV8	LV9	LV10	LV11	LV12
Mid-ventricular	Twist	2.47	2.67	2.44	1.73	2.19	1.76
		(0.92)	(0.94)	(1.23)	(1.63)	(1.00)	(0.94)
	No Twist	2.61	2.75	2.54	1.82	2.28	1.93
		(0.87)	(0.88)	(1.13)	(1.57)	(0.93)	(0.81)

		LV13	LV14	LV15	LV16		
Apical	Twist	3.45	3.26	2.25	1.85		
		(1.42)	(1.48)	(0.60)	(0.80)		
	No Twist	3.89	3.31	2.48	1.95		
		(1.20)	(1.36)	(0.77)	(0.78)		

The averages and standard deviations of RMS tracking errors across the eight Cine MR Datasets are given. Results are shown with and without the twisting in the deformable model.

**Table 5:** The summary of the results for the 16-point LV endocardium model.

Plane Location	Model	POI Index					
		Average RMS tracking errors [mm]					
		(Std. Dev.) [mm]					
		LV1	LV2	LV3	LV4	LV5	LV6
Basal	Twist	2.20	3.16	2.72	2.01	1.82	2.63
		(1.11)	(1.49)	(1.10)	(1.34)	(0.94)	(1.07)
	No Twist	2.35	3.27	2.83	2.20	1.92	2.86
		(1.16)	(1.76)	(1.13)	(1.31)	(1.06)	(1.19)

		LV7	LV8	LV9	LV10	LV11	LV12
Mid-ventricular	Twist	2.40	2.24	2.09	1.23	2.02	1.63
		(1.32)	(1.19)	(0.93)	(0.61)	(0.85)	(0.50)
	No Twist	2.64	2.31	2.17	1.65	2.21	2.05
		(1.37)	(0.95)	(0.96)	(0.62)	(0.87)	(0.88)

		LV13	LV14	LV15	LV16		
Apical	Twist	2.89	2.26	1.58	1.69		
		(1.38)	(0.99)	(0.55)	(0.83)		
	No Twist	3.04	2.40	1.95	1.86		
		(1.45)	(1.09)	(0.53)	(0.82)		

The averages and standard deviations of RMS tracking errors across the eight Cine MR datasets are given. Results are shown with and without the twisting in the deformable model.

**Table 6:** The summary of the results for the 8-point RV model.

Plane Location	Model	POI Index		
		Average RMS Tracking Errors [mm]		
		(Std. Dev.) [mm]		
		RV1	RV2	RV3
Basal	Twist	2.43 (0.95)	2.76 (1.21)	3.02 (1.32)
	No Twist	2.48 (0.96)	2.93 (1.40)	3.36 (1.16)

		RV4	RV5	RV6
Mid-ventricular	Twist	2.30 (1.27)	2.07 (0.60)	2.82 (1.06)
	No Twist	2.63 (1.18)	2.14 (0.62)	3.26 (1.25)

		RV7	RV8	
Apical	Twist	3.11 (1.15)	3.65 (1.36)	
	No Twist	3.60 (1.39)	5.18 (1.72)	

The averages and standard deviations of RMS tracking errors across the eight Cine MR datasets are given. Results are shown with and without the twisting in the deformable model.

The presented results show that torsional component is an essential part of the LV motion and integrating it to the parameterized deformations provide higher tracking accuracy. Without incorporating the twisting motion, the RMS errors are respectively 2.52 mm for the POIs on LV and 3.20 mm for the POIs on RV. The overall RMS error is 2.61 mm. The relative improvement in the tracking accuracy across the POIs are different when the torsion is included in the parameterized deformations. This further shows that the deformations are changing throughout the surface.

The results for the second experiment set are given in Table 7. As the slice planes were allowed to change any time step, the POIs at each time would be different unless the current slice was randomly chosen to be same as the previous one. For LV and RV, respectively 12 (6 for each epicardium and endocardium) and 3 POIs chosen per time step (Fig. 8b). Table 7 summarizes the results for the varying slice plane experiments. First, the RMS tracking errors of the all POIs tracked during the data duration are computed for each of the eight datasets. Then, the averages of these RMS tracking errors across all the eight datasets are calculated and reported together with the corresponding standard deviations.

The results show that with the varying slice plane, the tracking errors are within 3 pixels (5.3 mm). The RMS errors are respectively 3.12 mm for the POIs located on LV and 3.71 mm for the POIs located on RV. The overall RMS error is 3.42 mm. The proposed algorithm was able to track the POIs on LV more accurately then the POIs on RV, indicating proposed parameterization captures the deformations for the LV surface better compared to the RV surface.

**Table 7:** The summary of the results for the varying slice plane experiments.

	Location of POIs		
	LV Epi	LV Endo	RV
Mean RMSE (Std. Dev) [mm]	2.99 (1.33)	3.25 (1.40)	3.71 (1.87)

The averages and standard deviations of the RMS tracking errors across the eight Cine MR datasets are presented.

## Discussion

This work serves a proof-of-concept study for modeling and tracking the cardiac surface deformations via a low-order probabilistic model. The feasibility of the algorithm is shown with simulations and real cardiac datasets. For the real cardiac MRI datasets, where each dataset represents a single cardiac cycle, the presented method was able to track the POIs located on different sections of the cardiac surface within 3 pixels of accuracy. For the real cardiac MR dataset, the RMS tracking errors are respectively 2.61 mm for the fixed image slice plane and 3.42 mm for the varying image slice plane.

**Figure 3:**
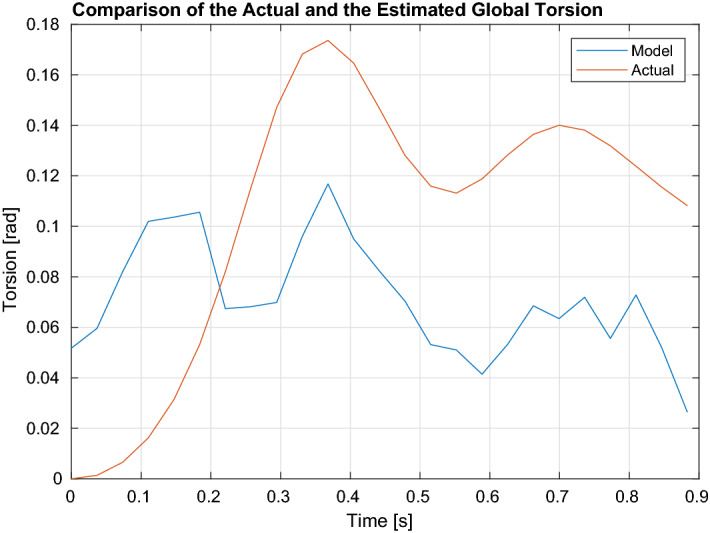
Shows the comparison of global torsion through the cardiac cycle estimated via the Segment software and the proposed algorithm. The mean estimation error is 0.06 rads.

One of the reasons for using the biventricular deformable model in the proposed method was to utilize the relative location of RV with respect to LV during the cardiac cycle to approximate the information regarding the twisting motion. As myocardium appears homogeneous in the Cine MR images, Cine MRI does not provide sufficient information regarding torsional component of the LV motion. Figure 3 shows the comparison of global torsion through the cardiac cycle estimated via Segment software and the algorithm.

It can be observed that initially the proposed method overshoots the estimation of the torsional component. Yet, as the transient period settles, it was able to follow the pattern of the twisting motion given by the software. The mean torsion estimation error is 0.06 rads (3.7 degs). Incorporating the twisting motion yielded 0.2–0.3 mm of improvement in the tracking accuracy. This improvement together with the cardiac surface tracking accuracy around 2.6 mm could pave the way for achieving the clinically-desired instrument to target accuracy of less than 3 mm during targeted intracardiac ablation procedures under image guidance, given the ablation catheter can be manipulated with enough precision^75^.

As this work focused on the feasibility of the presented method, the computational performance was not a priority. For a tractable on-line implementation, considerable speedup could be achieved by using a faster programming language than MATLAB and parallelization. The computation time could be reduced by performing further bench-top experiments to perform an extensive analysis of the system state.

The order of the adaptive filters is selected empirically based on the data duration. The higher the filter order, the more past samples it uses for the one-step prediction. This would result in monotonically decreasing one-step prediction error in magnitude because if the new filter weights, due to the additional filter order, were held to be zero, the same error of the lower order case would be obtained. Yet, storing more past samples and utilizing more information come at the expense of computational load. A more detailed discussion regarding this trade-off is given in^54^.

The on-line real-time execution of the cardiac surface tracking also requires substantial engineering work for improving the real-time image acquisition and reconstruction^76^ as well as the low-level interfacing to the hardware of the MRI scanner, to be able to control the imaging parameters on-line. This system integration work was outside the scope of the present study and has been left for future work.

In this work, multiple real cardiac MR datasets representing the right and left ventricle motion were used for evaluation. As such, the order of the parameterizations used to model the cardiac surface is influenced by the given datasets. Further testing of the presented approach with additional datasets; *i*.*e*.  atrium, tagged MRI, has been left for future work.

Using presegmented image slices was one of the assumptions made in this work. On-line segmentation of the measured image slices also needs to be handled for a real-time execution of the proposed algorithm. As this work focused on surface tracking and not segmentation, the on-line segmentation aspect of the framework will be addressed in the future.

The comparison of the presented approach with other methods remains future work. One interesting approach would be employing least squares Levenberg-Marquardt estimation in each of the time steps, in addition to the initialization as it was used in this study and evaluating the tracking performance of such a detection based approach.

Accounting for the positional offsets between the 2D slices that might occur during the cardiac multi-slice cine MRI data collection is outside the scope of the presented study. Previous studies investigated this problem^77^, which would be another avenue to explore as a future work.

In the estimation problem given in (1), the measurement model (48) is a nonlinear function of the state. Aside from the nonparametric particle filtering approach employed in this paper, the Extended Kalman filter (EKF) and the unscented Kalman filter (UKF) are two Gaussian techniques that could be applied to solve such nonlinear systems, where the beliefs (2) and (3) are represented by multivariate normal distributions^78^. Since 2D segmented binary images are used as measurements in this study, the measurement update step would primarily influence the computational and memory requirements of these Kalman filtering approaches due to the necessary matrix inversion^79^. Even for a small $100 \times 100$ pixels image, this would require a $10,000 \times 10,000$ matrix to be inverted. Given the datasets used in the presented work (Table 10) include much larger images, this would be an onerous task. In the case of UKF, the matrix inversion needs to be repeated for each sigma point^79^, making it even more demanding. Additionally, EKF requires the computation of Jacobians^79^, which would be very challenging for the presented highly nonlinear measurement model (46) to (49). For these reasons, the particle filtering approach is utilized in this study to solve the estimation problem in (1). In^80^, authors applied a dual Kalman filtering technique to the beating heart tracking problem to overcome the drawbacks of EKF, where 1D principle component signals are used as measurements that are extracted from 3D motion signals.

The machine and deep learning based methods are other potential avenues to explore for the cardiac surface tracking problem^81^. The data used in this study would be limited to utilize these approaches for the presented work. It remains a future work to adopt these schemes with substantial additional data and compare with the presented approach in this paper.

## Methods

### Problem formulation

This section explains the proposed method to solve the cardiac surface tracking problem.

The cardiac surface motion is modeled as a stochastic dynamic system. System state is specified by a probability distribution, which is defined over all the possible values that it can take. Consequently, the surface tracking problem is formulated as an estimation of the posterior distribution of the system state at each time step based on all observed data. A Bayesian approach is utilized for recursively estimating the posterior distribution from MR images.

Let $x_t$ be the dynamic system state that fully describes the cardiac motion and deformation at time *t*. Let $z_t$ be the measurement at time *t* and ${z}_{1:t} = \left[ z_1,z_2,\ldots ,z_t\right]$ represent the all observed data up till time *t*. The tracking problem is estimating the posterior distribution of the state conditioned on the available data; $b(x_t) = p({x}_t | {z}_{1:t})$, which is denoted as the belief of the dynamic system about its current state.

Using Bayes’ theorem, belief distribution becomes^78^:

1 $$\begin{aligned} \begin{aligned} p({x}_t | {z}_{1:t})&= \frac{p(z_t | x_t,z_{1:t-1})p(x_t | z_{1:t-1})}{p(z_{1:t-1})}\\&= \eta p(z_t | x_t)p(x_t | z_{1:t-1}), \end{aligned} \end{aligned}$$

where it is presumed that state $x_t$ is complete under Markov assumption; *i*.*e*., given $x_t$ past measurement convey additional information on predicting $z_t$^78^. In (1), $p(z_t | x_t)$ is the measurement model, which describes the likelihood of the measurement given current system state, $\bar{b}_t = p(x_t | z_{1:t-1})$ is the prediction distribution, which is the belief before incorporating $z_t$, and $\eta$ is a normalization constant ensuring final multiplication is a probability. Rewriting $\bar{b}_t$ via marginalization property:

2 $$\begin{aligned} \bar{b}_t&= \int p(x_t, x_{t-1} | z_{1:t-1})d x_{t-1} \nonumber \\&= \int p(x_t | x_{t-1}, z_{1:t-1})p(x_{t-1} | z_{1:t-1})d x_{t-1} \nonumber \\&= \int p(x_t | x_{t-1})b_{t-1}d x_{t-1}. \end{aligned}$$

In the last line of (2), Markov assumption for the state $x_{t-1}$ is again exploited; *i*.*e*., given the past state $x_{t-1}$, the present state $x_{t}$ is conditionally independent of the past measurements^78^. In (2), $p(x_t | x_{t-1})$ is the motion model which describes the stochastic dynamics of the system. Using (2) in (1) gives:

3 $$\begin{aligned} \begin{aligned} b_t = \eta p(z_t | x_t)\int p(x_t | x_{t-1})b_{t-1}d x_{t-1}. \end{aligned} \end{aligned}$$

(3) describes the Bayes filter, which recursively estimates the belief at time *t* from the belief at time $t-1$.

Bayes filter can be implemented in several ways depending on the approximations employed regarding the representations of motion and measurement models (linear/nonlinear) and belief distributions (Gaussian/nonparametric). Particle filters are used in this study for the implementation, which represent the posterior $b(x_t)$ by a finite set of random state samples drawn from this posterior^79^.

The particle filter algorithm is given in Algorithm 1. The input of the algorithm is the most recent measurement $z_t$ and the particle set $\mathscr{X}_{t-1}:= x_{t-1}^{[1]},x_{t-1}^{[2]},\cdots ,x_{t-1}^{[N_s]}$ representing the posterior $b(x_{t-1})$ at the previous time step $t-1$, where each particle $x_{t-1}^{[m]}$ with $m={1,2,\cdots ,N_s}$ is representing an instance of the state at time $t-1$ and $N_s$ denotes the total number of particles in the set $\mathscr{X}_{t-1}$.

In Algorithm 1, in order to construct the particle set $\mathscr{X}_{t}$ from the set $\mathscr{X}_{t-1}$, initially a temporary particle set ${\bar{\mathscr{X}}}_{t-1}$ is constructed via generating the hypothetical state $x_t^{[m]}$ from the particle $x_{t-1}^{[m]}$ based on the motion model (Line 4). Then, for each particle $x_t^{[m]}$ an importance factor $w_t^{[m]}$ is calculated based on the measurement model; yielding a weighted particle set (Line 5). Finally, the algorithm performs the resampling step, where it draws with replacement $N_s$ particles from the set ${\bar{\mathscr{X}}}_{t-1}$. The probability of drawing each particle is given by its importance weight. After the resampling step, the final particle set $\mathscr{X}_t$ is distributed according to the posterior $b_t$ in (3). Various options exist for the resampling step. A low-variance resampler is used in this study^78^.
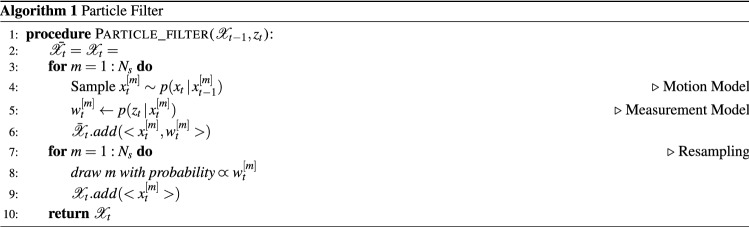


### Dynamic system state

Cardiac motion is a combination of rigid motion and deformations. The deformation space is infinite-dimensional. A low-dimensional parameterization of this space is needed for a tractable implementation of the particle filter. Deformable surface models are chosen for the representation of cardiac motion. This section briefly introduces these models and their adaptation to this study. A more thorough treatment of the deformable model framework is given in^35^.

**Figure 4:**
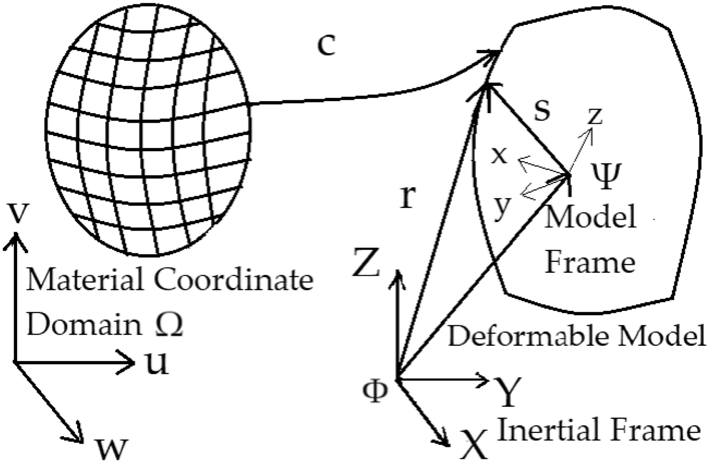
Visualizes the deformable model framework; mapping from the material coordinate domain to the deformable model.

In the deformable model framework, depicted in Fig. 4, the positions of the points on the deformed model *r* with respect to an inertial world coordinate frame $\Phi$ are given as:

4 $$\begin{aligned} \begin{aligned} r(m) = c + Rs(m), \end{aligned} \end{aligned}$$

where *s* is the reference shape of the model, describing the positions of points on the deformed model with respect to a model coordinate frame $\Psi$. *c* and *R* are the global translation and rotation of the model. $m=(u,v,w)$ are the material coordinates of the model, defined on a domain $\Omega$, which represent each point on the model in its undeformed state.

The reference shape *s* is defined as:

5 $$\begin{aligned} \begin{aligned} s = \xi (e(m; \alpha _0,\alpha _1,\cdots ,\alpha _k); \beta _0,\beta _1,\cdots ,\beta _l) = \xi (e; \beta ). \end{aligned} \end{aligned}$$

Here, *e* is a geometric primitive; such as an ellipsoid, that represents the model shape parametrically in *m* and parameterized by the variables $\alpha _{i}$ with $i=0,\cdots ,k$ and is subject to the global deformation, $\xi$ which depends on the parameters $\beta _j$ with $j=0,\cdots ,l$. $\xi$ can be a composite function of several deformations, i.e. $\xi (e; \beta ) = \xi _n(\cdots \xi _2(\xi _1(e; \beta )))$. Let ${\beta }_{1:l} = \left[ \beta _1,\beta _2,\cdots ,\beta _l\right]$, then the vector of deformation parameters are defined as:

6 $$\begin{aligned} \begin{aligned} \delta _s = \left[ \beta _{1:l}\right] ^{T}. \end{aligned} \end{aligned}$$

Along with the rigid motion parameters, $\delta _r$, which describes the global rotation and translation, the complete motion of the model is represented by following vector:

7 $$\begin{aligned} \begin{aligned} \delta = \left[ \delta _r, \delta _s\right] ^{T}. \end{aligned} \end{aligned}$$

Rest of this section describes application of this framework to the biventricular cardiac modeling. The biventricular deformable model of the heart consists of LV endocardium and epicadium, and only RV endocardium. The reference shape *s* is a blended model and composed of several primitive parts, which are combined by the use of a blending function. Portions of component primitives are cut out and the selected portions are joined together to build the whole model.

The prolate spheroid is chosen as the component primitive:

8 $$\begin{aligned} \begin{aligned} e(m)&= \begin{pmatrix}e_1 \\ e_2 \\ e_3 \end{pmatrix} = \begin{pmatrix}\rho \sinh {\sigma } \cos {u} \cos {v}\\ \rho \sinh {\sigma } \cos {u}\sin {v} \\ \rho \cosh {\sigma } \sin {u} \end{pmatrix}, \end{aligned} \end{aligned}$$

where $\rho$ and $\sigma$ are respectively the fixed focal radius and the constant radius of the prolate sphere that define its size. Thus, they correspond to the variables $\alpha _{i}$’s (5) that parameterize the geometric primitive *e*. The material coordinates, $m=(u,v,w)$, represent the resulting prolate spheroidal coordinate system, with $u=latitude$ and $v=longitude$. *w* is the number of primitives, where $w=1$ is the LV epicardium, $w=2$ is the LV endocardium, and $w=3$ is the RV endocardium, respectively. The material coordinate domain $\Omega$ is defined as $u \in \left[ -\frac{\pi }{2}, \frac{\pi }{6}\right]$ from apex to the base of the ventricles and $v \in [-\pi , \pi )$.

The shapes of LV endocardium, $e_{LV}(u,v,1)$, and epicardium, $e_{LV}(u,v,2)$, are defined by the prolate sphreoid primitive in (8) directly with:

9 $$\begin{aligned} \begin{aligned} e_{LV} = \begin{pmatrix}e_{{epi}_{1}}\lambda +e_{{endo}_{1}}(1-\lambda ) \\ e_{{epi}_{2}}\lambda +e_{{endo}_{2}}(1-\lambda )\\ e_{{epi}_{3}}\lambda +e_{{endo}_{3}}(1-\lambda ) \end{pmatrix}, \end{aligned} \end{aligned}$$

where $\lambda =1$ describing the LV epicardium in (8) and $\lambda =0$ describing the LV endocardium.

**Figure 5:**
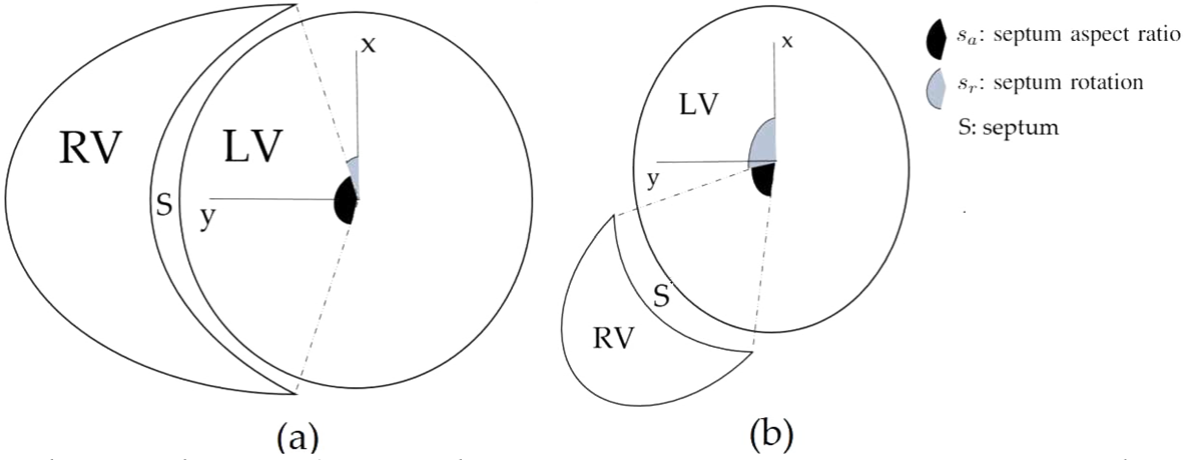
The planar shape of the RV relative to the LV for various septum aspect ratio $s_a$ and septum rotation $s_r$ values.

The RV endocardium is defined by a blended shape:

10 $$\begin{aligned} e_{RV}(u,v,3) = \left \{\begin{aligned} & e(u, s_a v + s_r,3)\; \;, \text {if}\; \; \;0\le v< \pi \\&e(u, - s_a v + s_r,3),\! \; \text {if}\; -\,\pi \le v < 0 \end{aligned}\quad ,\right. \end{aligned}$$

where $s_a$ is the arc length ratio of the septum with $0<s_a<1$ and $s_r$ is the angle between the end of the septum and the *x*-axis on the *xy*-plane with $0<s_r<\pi$ (Fig. 5).

The resulting blended biventricular shape model $e_B$ is:

11 $$\begin{aligned} e_B(m) = \left \{\begin{aligned}  &  e_{LV}(m)\;\text {if}\;w = 1,2 \\ & e_{RV}(m)\;\text {if}\;w = 3 \end{aligned}\quad .\right. \end{aligned}$$

Given the shape model, the ventricular deformations are defined by a set of volume preserving deformations (A volume preserving deformation conserves the volume of the object after the deformation. The mathematical description of the volume preserving deformations is provided in the Supplementary Material.)^70,82,83^. The parameters that describe these deformations are given in Table 8. These deformations are expressed by a set of functions; $f_i$’s for LV with $i=1,\cdots ,7$ and $h_i$’s for RV with $i=1,\cdots ,8$. They are applied sequentially to the geometric primitive to generate the reference shape *s* in (5). They describe how a point *P* is mapped from its undeformed reference position $p_0$ in the material domain $\Omega$ to its current deformed position $p_r$ in the model coordinate frame $\Psi$.

The nonrigid deformations are followed by a set of functions $G_j$’s with $i=j,\cdots ,4$, that describe the rigid body motion of the model with respect to an inertial world coordinate frame $\Phi$. They map the deformed position of the point $p_r \in \mathbb{R}^3$ from its model coordinate frame $\Psi$ to the position $p_s \in \mathbb{R}^3$ in the inertial world coordinate frame $\Phi$.

When describing the nonrigid motion of the cardiac surface via a set of deformations, the sequence of applying deformations is important. In this regard, the monotonically increasing numerical subscripts; *i*.*e*.  $i = 1\cdots ,n$, in the following equations refer to the intermediate position of the point $r_i \in \mathbb{R}^3$ after the deformation function $f_{i-1}$ is applied.

**Table 8:** Description of the parameterized deformations for left and right ventricles.

Ventricles	Deformation (function)	Parameter
LV	Radially dependent compression ($f_1$)	$k_1$
	Twisting along long axis ($f_2$)	$k_2 = s_r$
	Ellipticallization in long axis planes ($f_3$)	$k_3$
	Ellipticallization in short axis planes ($f_3$)	$k_4$
	Shear in *x* direction ($f_4$)	$k_5$
	Shear in *y* direction ($f_5$)	$k_6$
	Elongation in *z* direction ($f_6$)	$k_7$,$k_8$
RV	Radially dependent compression ($h_1$)	$l_1$
	Twisting along long axis ($h_2$)	$k_2 = s_r$
	Ellipticallization in long axis planes ($h_3$)	$l_2$
	Ellipticallization in short axis planes ($h_3$)	$l_3$
	Shear in *x* direction ($h_4$)	$l_4$,$l_5$
	Shear in *y* direction ($h_5$)	$l_6$,$l_7$
	Elongation in *x* direction ($h_6$)	$l_8$,$l_9$
	Elongation in *z* direction ($h_7$)	$l_{10}$,$l_{11}$
	Septum aspect ratio	$s_a$

Let $p_{L_0} = \left( p_{L_x}, p_{L_y}, p_{L_z}\right) ^{T}$ be a point on $e_{LV}$; the geometric primitive of LV. The subscripts *L* and 0 respectively state that point is located on the LV surface and it belongs to the initially undeformed reference position. After applying the function $f_0$, the point $p_{L_0}$ is mapped to a new one $r_1$.

It is desired that all the points on the surface of the prolate spheroid compress (or expand) uniformly. Yet, the points in the model can have different spherical radii and as a result nonuniform compression will occur in general. By converting the model to a more spherical shape, a uniform radial compression is obtained. In this regard, an initial transformation $f_0$ is applied to map a prolate spheroidal primitive into a more spherical one before applying the radially dependent compression deformation $f_1$:

12 $$\begin{aligned} \begin{aligned} r_1 = f_{0}(p_{L_0}) = \begin{pmatrix}\varsigma ^{\frac{1}{3}}_L p_{L_x}, \varsigma ^{\frac{1}{3}}_L p_{L_y}, \varsigma ^{\frac{-2}{3}}_L p_{L_z} \end{pmatrix}^{T}, \end{aligned} \end{aligned}$$

where the constant $\varsigma _L = \frac{\cosh {\sigma }}{\sinh {\sigma }}$ is a shape adjusting parameter for converting prolate spheroid primitive to a more spherical one. $\varsigma _L$ is given via calculating respective values for endocardium and epicardium, and then taking the average^38^.

The first mode of deformation $f_1$ is the radially dependent compression. It describes the change in LV chamber volume from the initial reference state by parameter $k_1$:

13 $$\begin{aligned} \begin{aligned} r_2&= f_{1}(r_1) \\&= \begin{pmatrix}\varepsilon _L r_{0_x}, \varepsilon _L r_{0_y}, \varepsilon _L r_{0_z} \end{pmatrix}^{T}, \end{aligned} \end{aligned}$$

with,

14 $$\begin{aligned} \begin{aligned} \varepsilon _L = \root 3 \of {1+\frac{3k_{1}V_{w_L}}{4\pi |r_1|^3}} , \end{aligned} \end{aligned}$$

where the $V_{w_L}$ is the wall volume of the model and $|r_1| = \sqrt{{r^2_{1_x}} + {r^2_{1_y}} + {r^2_{1_z}}}$.

The second deformation mode $f_2$ is the twisting motion of the ventricles and parameterized by $k_2$. Following the discussion in  “Introduction” section, the twisting motion of ventricles is captured by the septum rotation parameter $k_2 = s_r$:

15 $$\begin{aligned} \begin{aligned} r_3&= f_{2}(r_2) \\&= \begin{pmatrix}\cos ({\frac{\varsigma _L k_2 r_{2_z}}{|r_2|}})r_{2_x} - \sin ({\frac{\varsigma _L k_2 r_{2_z}}{|r_2|}})r_{2_y} \\ \sin ({\frac{\varsigma _L k_2 r_{2_z}}{|r_2|}})r_{2_x} + \cos ({\frac{\varsigma _L k_2 r_{2_z}}{|r_w|}})r_{2_y} \\ r_{2z} \end{pmatrix}, \end{aligned} \end{aligned}$$

where,

16 $$\begin{aligned} \begin{aligned} |r_2| = \sqrt{{r^2_{2_x}} + {r^2_{2_y}} + {r^2_{2_z}}} . \end{aligned} \end{aligned}$$

The next deformation mode $f_3$ determines if the shape of short-axis and long-axis cross-sections of the LV becomes more elliptical or spherical. The short-axis and long-axis ellipticallizations are parametrized respectively by $k_3$ and $k_4$:

17 $$\begin{aligned} \begin{aligned} r_4 = f_{3}(r_3) = \begin{pmatrix}\varsigma ^{\frac{-1}{3}}_L e^{k_4-(\frac{k_3}{2})} r_{3_x} \\ \varsigma ^{\frac{-1}{3}}_L e^{-k_4-(\frac{k_3}{2})}r_{3_y} \\ \varsigma ^{\frac{2}{3}}_L e^{k_3}r_{3_z} \end{pmatrix}. \end{aligned} \end{aligned}$$

The function $f_3$ also reverses the effect of $f_0$; mapping the spherical shape into a prolate spheroidal one.

The set of deformations $f_4$ and $f_5$, parameterized respectively by $k_5$ and $k_6$, are the shears in the short-axis plane. They represent the shears respectively in the *x* and *y* coordinate planes:

18 $$\begin{aligned}{} & {} \begin{aligned} r_5 = f_{4}(r_4) = \begin{pmatrix}r_{4_x} + k_5r^{2}_{4_z}, r_{4_y}, r_{4_z} \end{pmatrix}^{T}. \end{aligned} \end{aligned}$$

19 $$\begin{aligned}{} & {} \begin{aligned} r_6 = f_{5}(r_5) = \begin{pmatrix}r_{5_x}, r_{5_y} + k_6r^{2}_{5_z}, r_{5_z} \end{pmatrix}^{T}. \end{aligned} \end{aligned}$$

The final nonrigid deformation mode $f_6$ represents the elongation of the model in the *z* coordinate plane and parameterized by $k_7$ and $k_8$. The elongation deformation is defined by viewing the three dimensional space as an infinite cascade of parallel planes. Then, each of these planes is translated along the normal direction instead of orthogonal to it. As the material elements that inhabit these planes are contracted or stretched in the normal direction, inverse operations must be performed in every one of the planes. In other words, if an element is stretched in one direction, then it must be contracted in an orthogonal direction to locally conserve volume^83^. In this case, it stretches the model in the *z* direction and compresses it in the *x* and *y* directions:

20 $$\begin{aligned} \begin{aligned} r_7 = f_{6}(r_6) = \begin{pmatrix}\frac{r_{6_x}}{\sqrt{g^\prime (r_{6_z})}}, \frac{r_{6_y}}{\sqrt{g^\prime (r_{6_z})}}, g(r_{6_z}) \end{pmatrix}^{T} , \end{aligned} \end{aligned}$$

where,

21 $$\begin{aligned} \begin{aligned} g(r_{6_z}) = k_7r_{6_z}^2 + k_8r_{6_z} , \end{aligned} \end{aligned}$$

and $g^\prime (\cdot )$ is the derivative of $g(\cdot )$.

After applying the sequence of deformations $f_i$’s, the point on the LV surface is mapped to its deformed configuration $r_7$ in the model coordinate frame $\Psi$ from its undeformed reference position $p_{L_0}$ in the material domain $\Omega$.

The rigid body motion of the model with respect to an inertial world coordinate frame $\Phi$ is expressed by the set of functions $G_j$’s with $j \in {1\cdots ,4}$. They map the position of the point from its model frame $\Psi$ to the inertial frame $\Phi$:

22 $$\begin{aligned} \begin{aligned} r_L = f_R(r_7) = \left( r_{L_x}, r_{L_y}, r_{L_z}\right) ^{T}, \end{aligned} \end{aligned}$$

where $r_L \in \mathbb{R}^3$ is the current deformed position of the point with respect to the inertial frame $\Phi$ and $f_R$ is a composite function representing the rigid body motion, where the functions $G_j$’s are applied sequentially:

23 $$\begin{aligned} \begin{aligned} f_R(r_7) = G_4(G_3(G_2(G_1(r_7)))). \end{aligned} \end{aligned}$$

Here, $G_{1}$ conveys the rotation about *x* axis by parameter $g_1$:

24 $$\begin{aligned} \begin{aligned} r_8 = G_{1}(r_7) = \begin{pmatrix}r_{7_x} \\ \cos {(g_1)}r_{7_y} - \sin {(g_1)}r_{7_z} \\ \sin {(g_1)}r_{7_y} + \cos {(g_1)}r_{7_z} \end{pmatrix} . \end{aligned} \end{aligned}$$

$G_{2}$ expresses the rotation about *y* axis by parameter $g_2$:

25 $$\begin{aligned} \begin{aligned} r_9 = G_{2}(r_8) = \begin{pmatrix}\cos {(g_2)}r_{8_x} + \sin {(g_2)}r_{8_z} \\ r_{8_y} \\ -\sin {(g_2)}r_{8_x} + \cos {(g_2)}r_{8_z} \end{pmatrix} . \end{aligned} \end{aligned}$$

$G_{3}$ expresses the rotation about *z* axis by parameter $g_3$:

26 $$\begin{aligned} \begin{aligned} r_{10} = G_{2}(r_9) = \begin{pmatrix}\cos {(g_3)}r_{9_x} - \sin {(g_3)}r_{9_y} \\ \sin {(g_3)}r_{9_x} + \cos {(g_3)}r_{9_y} \\ r_{9_z} \end{pmatrix} . \end{aligned} \end{aligned}$$

Finally, $G_{4}$ expresses the translations in *x*, *y*, and *z* axes and parameterized by $g_4$, $g_5$, and $g_6$:

27 $$\begin{aligned} \begin{aligned} r_{L} = G_{4}(r_{10}) = \begin{pmatrix}r_{10_x} + g_4, r_{10_y} + g_5, r_{10_z} + g_6 \end{pmatrix}^{T} , \end{aligned} \end{aligned}$$

where as in (22), $r_L$ is the current deformed position of the point on LV surface with respect to the inertial frame $\Phi$.

Applying the deformable framework to the points on the RV surface follows the same approach.

Let $p_{R_0} = [p_{R_x}, p_{R_y}, p_{R_z}]^{T} \in \mathbb{R}^3$ be a point on $e_{RV}$; the geometric primitive of RV. The subscripts *R* and 0 respectively state that point is located on the RV surface and it belongs to the initially undeformed reference position.

The deformations applied sequentially to map the initially undeformed position of the point in the material domain $\Omega$ to the current position in the model frame $\Psi$. The monotonically increasing numerical subscripts; *i*.*e*.  $i = 1\cdots ,n$, in the following equations refer to the intermediate position of the point $q_i \in \mathbb{R}^3$ after the $h_{i-1}$ is applied. After applying the initial function $h_0$, the point $p_{R_0}$ is mapped to a new one $q_1$.

Likewise in the LV model, an initial transformation $h_0$ is applied to RV model to map a prolate spheroidal primitive into a more spherical one before applying the radially dependent compression deformation $h_1$ so that a uniform radial compression is obtained:

28 $$\begin{aligned} \begin{aligned} q_1 = h_{0}(p_{R_0}) = \begin{pmatrix}\varsigma ^{\frac{1}{3}}_R p_{R_x}, \varsigma ^{\frac{1}{3}}_R p_{R_y}, \varsigma ^{\frac{-2}{3}}_R p_{R_z} \end{pmatrix}^{T}, \end{aligned} \end{aligned}$$

where the constant $\varsigma _R$ is a shape adjusting parameter.

The first mode of deformation $h_1$ is the radially dependent compression parameterized by $l_1$. It describes the change in RV chamber volume from the initial reference state:

29 $$\begin{aligned} \begin{aligned} q_2 = h_{1}(q_1) = \begin{pmatrix}\varepsilon _R q_{1_x}, \varepsilon _R q_{1_y}, \varepsilon _R q_{1_z} \end{pmatrix}^{T}, \end{aligned} \end{aligned}$$

with,

30 $$\begin{aligned} \begin{aligned} \varepsilon _R = \root 3 \of {1+\frac{3l_{1}V_{R}}{4\pi |q_1|^3}} . \end{aligned} \end{aligned}$$

where $V_{R}$ is the volume of the model and $|q_1| = \sqrt{{q^2_{1_x}} + {q^2_{1_y}} + {q^2_{1_z}}}$.

The second deformation mode $h_2$ is the twisting motion of the ventricles. Following “Introduction” section, assuming ventricles have similar twisting patterns and letting $k_2 = s_r$:

31 $$\begin{aligned} \begin{aligned} q_3&= h_{2}(q_2) \\&= \begin{pmatrix}\cos ({\frac{\varsigma _R s_r q_{2_z}}{|q_2|}})q_{2_x} - \sin ({\frac{\varsigma _R s_r q_{2_z}}{|q_2|}})q_{2_y} \\ \sin ({\frac{\varsigma _R s_r q_{2_z}}{|q_2|}})q_{2_x} + \cos ({\frac{\varsigma _R s_r q_{2_z}}{|q_2|}})q_{2_y} \\ q_{2_z} \end{pmatrix}, \end{aligned} \end{aligned}$$

where,

32 $$\begin{aligned} \begin{aligned} |q_2| = \sqrt{{q^2_{2_x}} + {q^2_{2_y}} + {q^2_{2_z}}} . \end{aligned} \end{aligned}$$

The next deformation mode $h_3$ determines the short-axis and long-axis ellipticallizations of the RV and are respectively parametrized by $l_2$ and $l_3$:

33 $$\begin{aligned} \begin{aligned} q_4 = h_{3}(q_3) = \begin{pmatrix}\varsigma ^{\frac{-1}{3}}_R e^{l_3-(\frac{l_2}{2})} q_{3_x} \\ \varsigma ^{\frac{-1}{3}}_R e^{-l_3-(\frac{l_2}{2})}q_{3_y} \\ \varsigma ^{\frac{2}{3}}_R e^{l_2}q_{3_z} \end{pmatrix}. \end{aligned} \end{aligned}$$

The function $h_3$ also reverses the effect of $h_0$; mapping the spherical shape into a prolate spheroidal one.

The set of deformations $h_4$ and $h_5$ represent the shears respectively in the *x* and *y* coordinate planes. The function $h_4$ is parameterized by $l_4$ and $l_5$ and the function $h_5$ is parameterized by $l_6$ and $l_7$:

34 $$\begin{aligned}{} & {} \begin{aligned} q_5 = h_{4}(q_4) =\begin{pmatrix} q_{4_x} + l_4q^{2}_{4_y} + l_5q^{2}_{4_z} \\ q_{4_y} \\ q_{4_z} \end{pmatrix}. \end{aligned} \end{aligned}$$

35 $$\begin{aligned}{} & {} \begin{aligned} q_6 = h_{5}(q_5) =\begin{pmatrix} q_{5_x} \\ q_{5_y} + l_6q^{2}_{5_x} + l_7q^{2}_{5_z} \\ q_{5_z} \end{pmatrix}. \end{aligned} \end{aligned}$$

The final nonrigid deformation modes $h_6$ and $h_7$ represent the elongations of the model respectively in the *x* and *z* coordinate planes. The function $h_6$ is parameterized by $l_8$ and $l_9$. It stretches the model in the *x* direction and compresses it in the *y* and *z* directions. The function $h_7$ is parameterized by $l_{10}$ and $l_{11}$. It stretches the model in the *z* direction and compresses it in the *x* and *y* directions:

36 $$\begin{aligned} \begin{aligned} q_7 = h_{6}(q_6) = \begin{pmatrix} g(q_{6_x}), \frac{q_{6_y}}{\sqrt{g^\prime (q_{6_x})}}, \frac{q_{6_z}}{\sqrt{g^\prime (q_{6_x})}} \end{pmatrix}^{T} , \end{aligned} \end{aligned}$$

where,

37 $$\begin{aligned}{} & {} \begin{aligned} g(q_{6_x}) = l_8q_{6_x}^2 + l_9q_{6_x}. \end{aligned} \end{aligned}$$

38 $$\begin{aligned}{} & {} \begin{aligned} q_8 = h_{7}(q_7) = \begin{pmatrix} \frac{q_{7_x}}{\sqrt{g^\prime (q_{7_z})}}, \frac{q_{7_y}}{\sqrt{g^\prime (q_{7_z})}}, g(q_{7_z}) \end{pmatrix}^{T}, \end{aligned} \end{aligned}$$

where,

39 $$\begin{aligned} \begin{aligned} g(q_{7_z}) = l_{10}q_{7_z}^2 + l_{11}q_{7_z} . \end{aligned} \end{aligned}$$

After the sequence of deformations $h_i$’s are applied, the point on the RV surface is mapped to its deformed configuration $q_8$ in the model coordinate frame $\Psi$ from its undeformed reference position $p_{R_0}$ in the material domain $\Omega$.

Likewise in the LV model, the rigid body motion of the model with respect to an inertial world coordinate frame $\Phi$ is expressed by the set of functions $G_j$’s with $j \in {1\cdots ,4}$. They map the position of the point from its model coordinate frame $\Psi$ to the inertial frame $\Phi$:

40 $$\begin{aligned} \begin{aligned} r_R = f_R(q_7) = \left( r_{R_x}, r_{R_y}, r_{R_z}\right) ^{T}, \end{aligned} \end{aligned}$$

where $r_{{R}} \in \mathbb{R}^3$ is the current deformed position of the point on RV surface with respect to the inertial frame $\Phi$ and $f_R$ is the composite function representing the rigid body motion, given through (23) to (27).

Biventricular deformations are then described by the following 20-dimensional vector:

41 $$\begin{aligned} \delta _s = \left[ k_{1:8}, l_{1:11}, s_a\right] ^T , \end{aligned}$$

where $s_a$ and $k_2 = s_r$ are respectively the septum aspect ratio and septum rotation parameters from (10).

Rigid motion is described with the six parameters defined in (23), $\delta _r = \left[ g_{1:6}\right] ^T$. As a result, the dynamic system state $x_t$ is a 26-dimensional vector:

42 $$\begin{aligned} x_t = \left[ k_{1:8}, l_{1:11}, s_a, g_{1:6}\right] ^T , \end{aligned}$$

where the subscript *t* denoting the current time step is omitted on the right hand side of (42).

### Cardiac surface tracking

There are two key steps in the particle filter algorithm; the motion update step and the measurement update step.

The motion update given in (2) step propagates the belief from the previous time step $t-1$ based on the dynamic system model $p(x_t|x_{t-1})$ and performs the prediction. Dynamic system model specifies how the current system state $x_t$ evolves from the previous state $x_{t-1}$. As this dynamic system is stochastic, the process that models the state’s evolution is described by a probability distribution; $p(x_t|x_{t-1})$.

The measurement update given in (3) incorporates the observed data based on the measurement model $p(z_t|x_t)$, performs the correction, and computes the posterior belief distribution. The measurement model specifies how the measurement $z_t$ is generated from the state $x_t$. As the dynamic system is stochastic, the process that models this generation is a probability distribution; $p(z_t|x_{t})$. The next two sections describe these two steps in detail.

#### Motion update

Cardiac surface motion has complex dynamics and shows high variability, making it challenging to describe with an exact motion model. In^54^ cardiac dynamics are assumed to be generated by a vector-autoregressive (VAR) process, which is adapted in this work.

At each time step *t*, given the incremental state update of previous time step; ${\delta x_{t-1} = x_{t-1}-x_{t-2}}$, and a vector of past *N* increments; $\Delta x_{N_{t-2}} = [\delta x_{t-2}^T, \delta x_{t-3}^T,\cdots ,\delta x_{t-(N+1)}^T]^T$, a recursive least squared (RLS) based adaptive filter is used to estimate weights of the underlying VAR model:

43 $$\begin{aligned} \begin{aligned} \delta x_{t-1}&= W_{t-1} \Delta x_{N_{t-2}} \\&= W_{t-1}[\delta x_{t-2}^T, \delta x_{t-3}^T,\cdots ,\delta x_{t-(N+1)}^T]^T , \end{aligned} \end{aligned}$$

where the weight matrix $W_{t-1}$ is estimated such that the square of the error between the two sides of (43) is minimized. Once the weights are estimated, the vector of past *N* state increments is updated with the latest increment; $\Delta x_{N_{t-1}} = [\delta x_{t-1}^T, \delta x_{t-2}^T,\cdots ,\delta x_{t-N}^T]^T$. The estimated weights and the updated state increment vector are used to predict the current state update $\delta \hat{x}_t$ ($\delta \hat{x}$ denotes the predicted state increment, $\delta x$ is the actual increment calculated once the state is estimated.):

44 $$\begin{aligned} \begin{aligned} \delta \hat{x}_t = W_{t-1} \Delta x_{N_{t-1}} . \end{aligned} \end{aligned}$$

Then, motion model is given by:

45 $$\begin{aligned} \begin{aligned} \hat{x}_t = x_{t-1} + \delta \hat{x}_{t} + \nu _{t}, \end{aligned} \end{aligned}$$

where $\nu _{t}$ is the Gaussian process noise representing the model uncertainties with covariance $\Sigma _v$; $\nu _{t}\sim \mathscr{N}(0,\Sigma _{\nu })$, and $\hat{x}_t$ is the state estimate before incorporating measurement. A more thorough treatment of the state estimation based on RLS adaptive filters is given in^54^.

#### Measurement update

In this study measurements are the 2D segmented binary images, which are generated from the delineated boundaries of the ventricles. The delineated boundaries of the ventricles are represented as the zero-level set^32^ of the intersection of the cardiac surface and the image slice plane. Rest of this section explains this measurement model, which describes how a measurement is generated given the system state.

For a system state $x_t$, let the set of points on the cardiac surface (4) in $\mathbb{R}^3$ represented by $\gamma = \left[ \gamma _x, \gamma _y, \gamma _z\right]$ and let the arbitrarily oriented image slice plane be expressed by the general plane equation in $\mathbb{R}^3$:

46 $$\begin{aligned} \begin{aligned} a_px + b_py + c_pz = d_p \end{aligned} \end{aligned}$$

The signed distances of the surface points to the given image slice plane are calculated as:

47 $$\begin{aligned} \begin{aligned} \varphi (\gamma _x,\gamma _y,\gamma _y) = a_p\gamma _x + b_p\gamma _y + c_p\gamma _z - d_p \end{aligned} \end{aligned}$$

The zero-set of the signed distances gives the points on the surface, which represent the boundaries of the ventricles:

48 $$\begin{aligned} \begin{aligned} \Gamma : \{(\gamma _x,\gamma _y,\gamma _y)\;|\;\varphi (\gamma _x,\gamma _y,\gamma _y) = 0\}. \end{aligned} \end{aligned}$$

The 2D binary mask generated via the boundaries $\Gamma$ is the predicted measurement $\hat{z}_t$; giving the measurement model:

49 $$\begin{aligned} \begin{aligned} {z}_t&= \zeta (x_{t}, \omega _{t}) \\&= \hat{z}_t + \omega _{t} , \end{aligned} \end{aligned}$$

where the measurement function $\zeta$ represents the process of generating the predicted image $\hat{z}_t$ from the state $x_{t}$ and $\omega _t$ is the Gaussian measurement noise.

**Figure 6:**
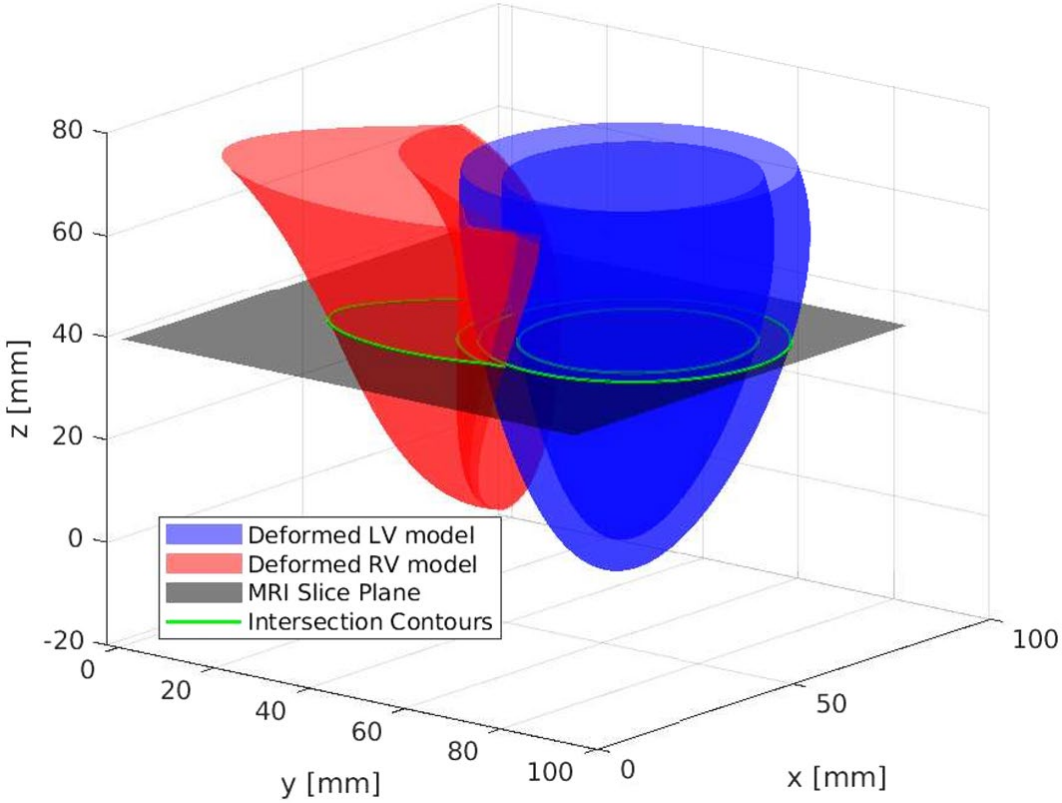
Shows the intersection of the biventricular deformable model and MRI slice plane.

**Figure 7:**
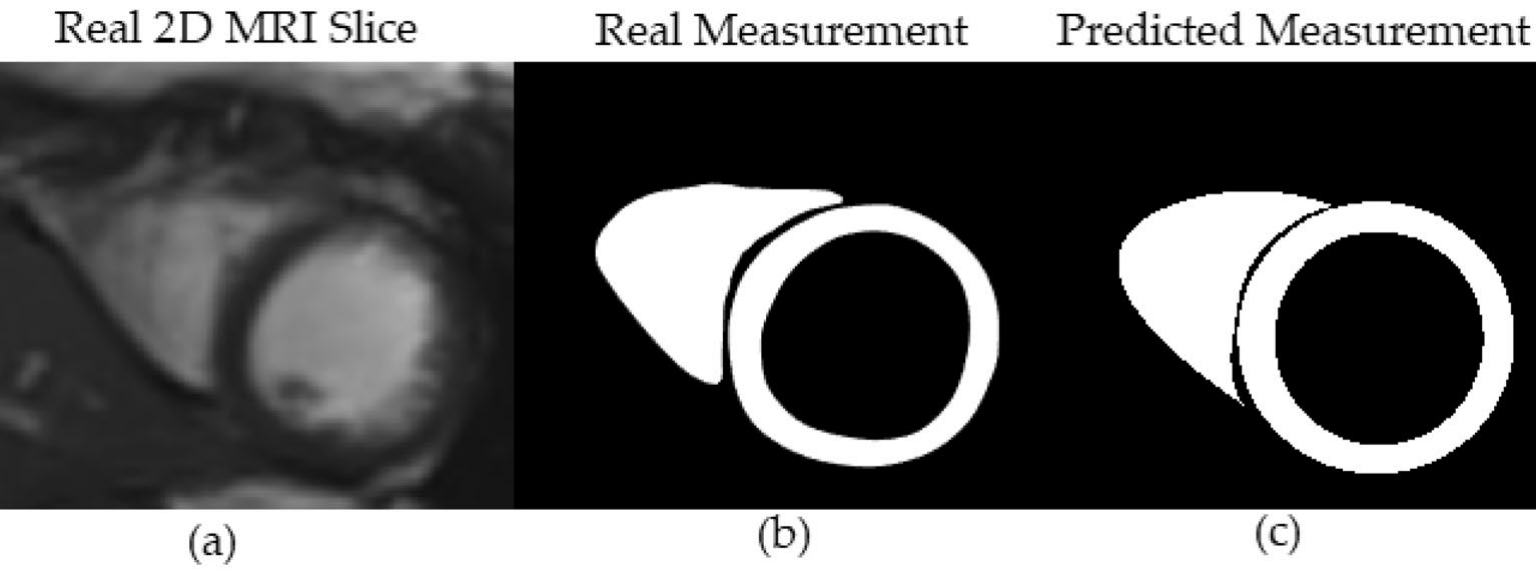
(**a**) The real cardiac MRI 2D image slice. (**b**) The segmented binary slice measurement $z_t$. (**c**) The predicted binary measurement $\hat{z}_t$, generated from the contours obtained via the intersection of deformable model and image slice plane.

Figure 6 shows the deformable model and image slice plane intersection. Figure 7a shows the real cardiac MRI 2D image slice. Figure 7b shows the corresponding segmented binary measurement $z_t$. Figure 7c shows the predicted binary image $\hat{z}_t$, generated from the contours obtained via the intersection of deformable model and image slice plane.

The measurement likelihood between the given segmented image and predicted image is calculated via normalized cross-correlation (NCCORR)^84^:

50 $$\begin{aligned} \begin{aligned} \kappa =\frac{\sum _{x,y}^{} (z(x,y) -\bar{z}_{u,v})(\hat{z}(x-u,y-u) -\hat{z})}{\sqrt{\sum _{x,y}^{} (z(x,y) -\bar{z}_{u,v})^2\sum _{x,y}(\hat{z}(x-u,y-u) -\bar{\hat{z}})^2}} \end{aligned} \end{aligned}$$

The complete deformable cardiac surface tracking algorithm is given in Algorithm 2. $\Delta \mathscr{X}_{N_{t-2}}$ and $\Delta \mathscr{X}_{N_{t-1}}$ are the set of past *N* increments for the whole particle set respectively at times steps $t-2$ and $t-1$.
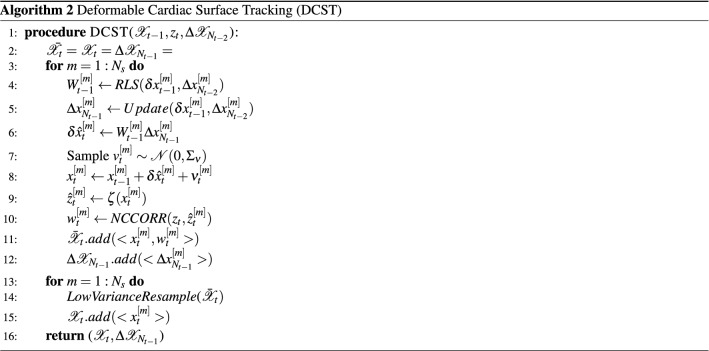


### Experimental methods

The proposed algorithm is implemented in MATLAB^®^ with offline analysis. The tracking step for a single particle takes approximately 0.12 seconds on an Intel^®^ 3.40GHz quad-core CPU with 16GB RAM under Linux operating system. For both the simulations and the experiments, the presented results are averaged across 5 Monte Carlo trials.

The study is not exempt from Institutional Review Board (IRB) approval. The approving institution is the University Hospitals Cleveland Medical Center (UHCMC) Institutional Review Board. The date of approval is 02/01/2018. The UHCMC IRB protocol number is 08-02-43 and all experiments and methods were performed in accordance with the relevant guidelines and regulations of this protocol. Informed consent was obtained from all subjects involved in the study.

#### Initialization

**Figure 8:**
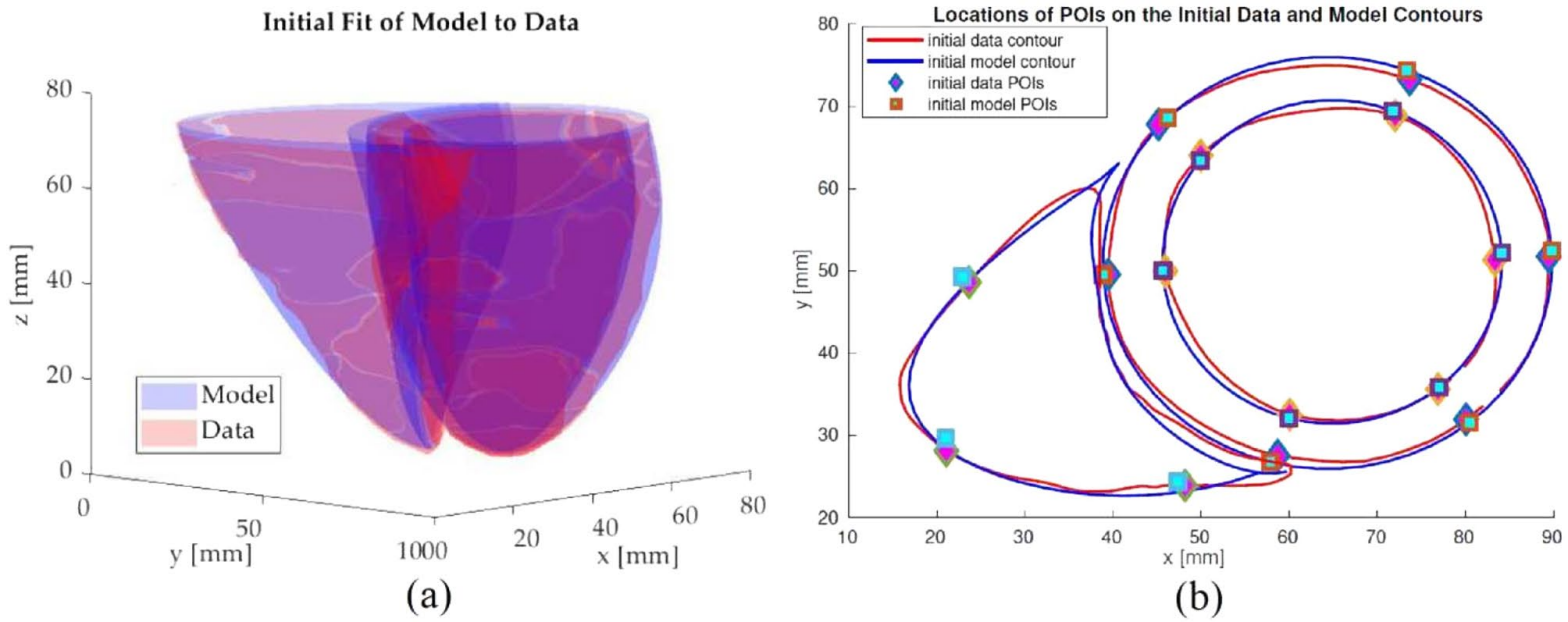
(**a**) Shows the initial fit of biventricular model to data via nonlinear least squares optimization. (**b**) Shows the locations of the initial points on the model and the data selected based on 16-point LV and 8-point RV model for a mid-ventricular slice.

For both the numerical phantom and the real cardiac MR datasets, the model is initialized at the first frame of the cardiac cycle via Levenberg-Marquardt nonlinear least-squares optimization method (Fig. 8a)^85^. Besides the initial deformation parameters, this step also provides the focal radius $\rho$ and constant radius $\sigma$ parameters (8) of the plorate spheroid primitive.

For the POIs selected via 16-point LV and 8-point RV models, their material coordinates are determined at the initialization step. Then, the corresponding points in the world coordinate frame for these material points are tracked over the cardiac cycle. Figure 8b shows the locations of initial points on the model and data for a mid-ventricular slice.

#### Numerical phantom

The numerical phantom allows to compare the performance of the proposed method with the precise ground truth. Quasi-periodic nature of the cardiac motion^54^ is utilized to construct the numerical phantom. Each parameter in Table 8 is initially generated from a sinusoidal signal for the duration of two periods and then added with the white Gaussian noise. The fundamental frequency and the sampling time of the signals are respectively chosen as $f_h=2$ Hz and $T_s=20$ ms to mimic cardiac heart rate^54^ and real-time MR multi-slice image acquisition^86^. The signals for LV parameters are adapted from the cardiac simulator presented in^82^. The RV parameters are assumed to vary similarly with LV. The parameter and septum aspect ratio (10) signals are given in Table 9.

**Table 9:** Parameter signals used to construct the numerical phantom.

Ventricles	Parameter	Signal
LV	*k*1	$0.01\sin {(2\pi f_h t)}$
	$k_2=s_r$	$\tfrac{\pi }{12}-\tfrac{\pi }{12}\cos {(2\pi f_h t)}$
	$k_3$	$-0.03\sin {(2\pi f_h t)}$
	$k_4$	$0.05\sin {(2\pi f_h t)}$
	$k_5$	$-0.02\sin (2\pi f_h t)$
	$k_6$	$0.02\sin (2\pi f_h t)$
	$k_7$,$k_8$	$0.01\sin (2\pi f_h t)$
RV	$l_1$	$0.01\sin {(2\pi f_h t)}$
	$l_2$	$-0.03\sin {(2\pi f_h t)}$
	$l_3$	$0.05\sin {(2\pi f_h t)}$
	$l_4$,$l_5$	$-0.02\sin (2\pi f_h t)$
	$l_6$,$l_7$	$0.02\sin (2\pi f_h t)$
	$l_8$,$l_9$	$0.01\sin (2\pi f_h t)$
	$l_{10}$,$l_{11}$	$0.01\sin (2\pi f_h t)$
	$s_a$	0.9 + 0.1$\cos {(2\pi f_h t)}$

$f_h=2$ Hz is the fundamental frequency and $T_s=20$ ms is the sampling time of the signals, where $t=nT_s$ with $n=\left[ 0,1\cdots ,49\right]$. Corresponding to a duration of two periods.

**Figure 9:**
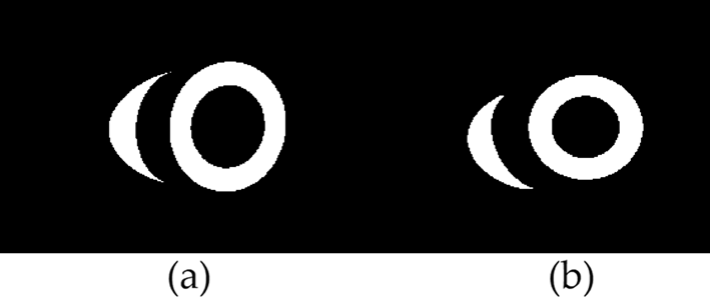
Slices from mid-ventricular section of the numerical phantom for (**a**) undeformed (**b**) deformed instances.

It is assumed that the numerical phantom is initially relaxed and undeformed, representing end-diastole. It starts to deform and reaches its maximum deformation at half signal period representing end-systole, then relaxes gradually to conclude a single signal period. Only nonrigid motion is considered for the numerical phantom. As the method can be generalized by adding rotation and translation parameters, tracking of the rigid motion is trivial. Image size is chosen to be 200x280 pixels with an in-plane resolution of 1.5x1.5 mm$^2$ and a slice thickness of 2 mm. Figure 9 shows respectively the undeformed and the deformed instances for a mid-ventricular slice.

For the numerical phantom, 10th order one-step estimators were used in the motion update step (45). The particle filter was initialized with 1000 particles per time step. Separate trials were performed for the basal, mid-cavity, and apical sections slice planes (Fig. 1a). In each trial once the initial 2D image slice plane was selected at the first time step, the same image slice was used in the remaining time steps.

#### Real cardiac MRI data

The experimental datasets used in this study are 2D multi-slice cardiac cine MRI sequences collected following standard CMR protocols^87^, where each dataset showing a single cardiac cycle divided into 25 cardiac phases. For all the datasets, the first time frame corresponds to diastole. Thus, each dataset starts at diastole, then continues to systole, and finally comes back to diastole. The in-plane resolution of each dataset is 1.77 × 1.77 mm$^2$ with slice thickness of 8 mm and no gap between slices. The information for each dataset about the number of slices covering from the base to the apex of the ventricles, the slice locations, and the image size of each slice is given in Table 10.

**Table 10:** Description of the 2D multi-slice cardiac cine MRI datasets used in the experimental validation.

Dataset	Image Size (pixels)	Number of Slices	Slice locations		
			Basal	Mid	Apical
1	204 × 243	8	{1,2,3}	{4,5,6}	{7,8}
2	188 × 192	9	{1,2,3}	{4,5,6}	{7,8,9}
3	211 × 297	9	{1,2,3}	{4,5,6}	{7,8,9}
4	284 × 294	8	{1,2,3}	{4,5,6}	{7,8}
5	243 × 268	9	{1,2,3}	{4,5,6}	{7,8,9}
6	331 × 322	8	{1,2,3}	{4,5,6}	{7,8}
7	293 × 328	9	{1,2,3}	{4,5,6}	{7,8,9}
8	284 × 291	8	{1,2,3}	{4,5,6}	{7,8}

For each dataset, the image size of a single slice, number of slices in that dataset, and the slice locations relative to basal, mid-ventricular, and apical sections are provided.

**Figure 10:**
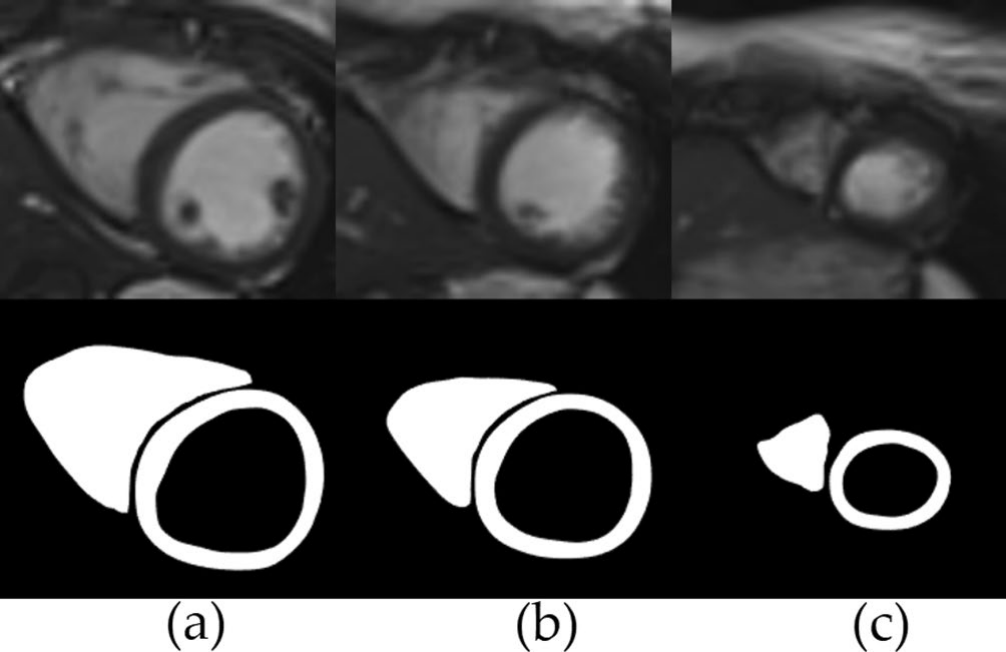
Shows the segmentations obtained via Segment software respectively for the (**a**) basal (**b**) mid-ventricular (**c**) apical slices.

The analysis of the cine MRI datasets were done offline via using the freely available software, Segment version 3.3 R9405b^88^. The LV and RV segmentations were performed by using the automatic segmentation algorithm in the software^89^. The ground truth points were extracted from the data via the feature tracking algorithm in the strain analysis module of the software^90^. Figure 10 shows the images and corresponding segmentations obtained via the Segment software for the basal, mid-ventricular, and apical slices.

For the real MRI datasets, 5th order one-step estimators were used in the motion update step. The particle filter was initialized with 1000 particles per time step. A single MRI slice was used per time step for the tracking algorithm.

Two sets of experiments were performed for the real cardiac MRI datasets. The first experiment was same as the numerical phantom. Separate trials were performed for the basal, mid-cavity, and apical slice planes. In each trial, once the 2D image slice plane was determined at the first time step, the same image was used in the remaining time steps.

In the second experiment, the algorithm was evaluated with changing slice planes at each time step; *i*.*e*., the slice plane was allowed to be different than the one in the previous time step. Once the initial 2D image slice plane was selected, it was not necessarily needed to be the same one in the remaining time steps. In a typical application, this varying single image slice could be either manually selected from a stack of slices by a clinician or automatically by an active sensing algorithm^91^. Here, the set of slice planes are selected randomly between the basal and mid-ventricular slices (Fig. 1). At each time step, the slice plane variable $v_p$; indicating which slice to be selected from a given stack of slices, is generated randomly from a discrete uniform distribution; $v_v\sim \mathscr{U}(v_b, v_m)$, where $v_b = 2$ is the second basal slice and $v_m = 7$ is the first apical slice.

## Supplementary Information

Supplementary Information.

## Data Availability

The datasets analysed during the current study are not publicly available due to restrictions in the Institutional Review Board approval. External researchers are welcome to contact the corresponding author (E. E. Tuna) for any inquiries about the data.
