# Supplementary material for: Deformable cardiac surface tracking by adaptive estimation algorithms
Source: Sci Rep. 2023 Jan 25;13:1387. doi: 10.1038/s41598-023-28578-0 (PMC9877032; doi:10.1038/s41598-023-28578-0)
Supplement: Supplementary file 1 — Supplementary Information. [file 41598_2023_28578_MOESM1_ESM.pdf]

SUPPLEMENTARY MATERIAL

Volume Preserving Deformations

Let a three dimensional object be described in Cartesian coordinates by $x = (x_1, x_2, x_3)^T$. A deformation is a mapping X transforms these coordinates into a new set of coordinates: $X = X(x) = (X_1, X_2, X_3)^T$. A volume preserving deformation is one that preserves the volume of the object after the deformation, which is mathematically described as:

$$\begin{aligned}\int_x dx_1 dx_2 dx_3 &= \int_X dX_1 dX_2 dX_3 \\ &= \int_x det(\nabla_x X) dx_1 dx_2 dx_3,\end{aligned} \tag{1}$$

where $\nabla_x X$ is the deformation gradient:

$$\nabla_x X = \begin{pmatrix} \frac{\partial X_1}{\partial x_1} & \frac{\partial X_1}{\partial x_2} & \frac{\partial X_1}{\partial x_3} \\ \frac{\partial X_2}{\partial x_1} & \frac{\partial X_2}{\partial x_2} & \frac{\partial X_2}{\partial x_3} \\ \frac{\partial X_3}{\partial x_1} & \frac{\partial X_3}{\partial x_2} & \frac{\partial X_3}{\partial x_1} \end{pmatrix}. \tag{2}$$

A locally volume preserving deformation is one that satisfies:

$$det(\nabla_x X) = 1. \tag{3}$$

Rigid body transformation of the form, $X(x) = Rx + r$, where $R \in SO(3)$, is an example of a locally volume preserving deformation.

A locally volume preserving deformation is also globally volume preserving; *i.e.*, (3) satisfies (1) certainly, but converse is not generally true. In addition, compositions of locally volume preserving deformations are also locally volume preserving. For instance, let $F(x)$ and $H(x)$ be two locally volume preserving deformations. Then, the compositions $H(F(x))$ and $F(H(x))$ are also locally volume preserving. This follows from the chain rule, *e.g.*:

$$\nabla_x F(H(x)) = \nabla_z F(z) \nabla_x H(x), \tag{4}$$

where $z = H(x)$. Following the fact that the determinant of product of matrices is the product of the determinants:

$$det(\nabla_x F(H(x))) = det(\nabla_z H(z)) det(\nabla_x H(x)) = 1 \tag{5}$$
